# Differential Localization of Pain-Related and Pain-Unrelated Neural Responses for Acupuncture at BL60 Using BOLD fMRI

**DOI:** 10.1155/2013/804696

**Published:** 2013-06-18

**Authors:** Na-Hee Kim, Seung-Yeon Cho, Geon-Ho Jahng, Chang-Woo Ryu, Seong-Uk Park, Chang-Nam Ko, Jung-Mi Park

**Affiliations:** ^1^Department of Cardiology and Neurology of Korean Medicine, College of Korean Medicine, Kyung Hee University, Seoul, Republic of Korea; ^2^Department of Radiology, Kyung Hee University Hospital at Gangdong, School of Medicine, Kyung Hee University, Seoul, Republic of Korea; ^3^Stroke and Neurological Disorders Center, Kyung Hee University Hospital at Gangdong, 149 Sangil-dong, Gangdong-gu, Seoul 134-727, Republic of Korea

## Abstract

The objective of this study was to differentiate between pain-related and pain-unrelated neural responses of acupuncture at BL60 to investigate the specific effects of acupuncture. A total of 19 healthy volunteers were evaluated. fMRI was performed with sham or verum acupuncture stimulation at the left BL60 before and after local anesthesia. To investigate the relative BOLD signal effect for each session, a one-sample *t*-test was performed for individual contrast maps, and a paired *t*-test to investigate the differences between the pre- and post-anesthetic signal effects. Regarding verum acupuncture, areas that were more activated before local anesthesia included the superior, middle, and medial frontal gyri, inferior parietal lobule, superior temporal gyrus, thalamus, middle temporal gyrus, cingulate gyrus, culmen, and cerebellar tonsil. The postcentral gyrus was more deactivated before local anesthesia. After local anesthesia, the middle occipital gyrus, inferior temporal gyrus, postcentral gyrus, precuneus, superior parietal lobule, and declive were deactivated. Pre-anesthetic verum acupuncture at BL60 activated areas of vision and pain transmission. Post-anesthetic verum acupuncture deactivated brain areas of visual function, which is considered to be a pain-unrelated acupuncture response. It indicates that specific effects of acupoint BL60 are to control vision sense as used in the clinical setting.

## 1. Introduction

Acupuncture is a widespread component of traditional medicine in Korea, China, and Japan and is characterized by stimulation via the insertion of needles, usually inducing some degree of pain. By noxious or external pressure sensation, the most commonly activated brain regions include the primary (S1) and secondary somatosensory (S2) cortices, as well as the prefrontal, anterior cingulate, temporal, and insular cortices [1, 2]. These cortices of ascending somatosensory pathways of pain-related activation are due to the transmission of noxious stimuli along the spinohypothalamic pathway, the spinopontoamygdaloid pathway, and the spinothalamic pathway [3–5].

Functional magnetic resonance imaging (fMRI) has been used to indirectly detect brain functions [6, 7]. Several human fMRI studies have also shown that acupuncture needling affects brain activation. The relationship between acupoint and correspondent brain location has been discussed [8, 9], and different methods of stimulation such as tactile acupuncture or superficial pricking are associated with specific brain activation [10, 11]. These results of neuronal activation suggest that acupuncture may be related to autonomic, cognitive, and affective functions, as well as sensorimotor control with connections to the cerebrum, brainstem, and other areas [8, 9, 11–13].

Acupuncture-evoked neuronal stimulation still remains unclear because acupuncture is composed of various components including pain or pressure sensation, as well as placebo effect from anticipation, imagination, or concentration. In order to investigate the “specific” effect of either penetrating or nonpenetrating acupuncture, it is crucial to determine whether and to what extent the effect is related to a pain response. 

Recently, fMRI studies have investigated acupoint at BL60 in a canine model [14]. Several fMRI studies using acupuncture at BL60 showed brain activation [15, 16], but, as mentioned earlier, the results did not distinguish whether the activated areas were related or unrelated to pain responses. To the best of our knowledge, no human studies have been published on fMRI activation in response to acupuncture before and after local anesthesia (i.e., with or without pain). Besides, there were some limitations from the previous canine model study [14]. We could not determine whether the dogs felt Deqi sensation and complete suppression of pain was uncertain. 

Localization of pain-related and pain-unrelated responses to acupuncture is necessary in order to discover acupuncture's effect on pain sensation. The objective of this study was to determine the nature of pain related and pain unrelated in order to define specific neural responses to acupuncture using acupoint BL60.

## 2. Materials and Methods

### 2.1. Study Subjects

Twenty female subjects (mean age: 29.5 ± 5.5 (SD) years; range: 21 to 43 years) consented to participate in this study. The inclusion criteria were as follows: (i) subjects had no past medical history or current history of physical or psychiatric diseases, (ii) they did not take and were currently not taking any prescribed or illicit drugs, and (iii) all subjected were right handed. They had experienced acupuncture. For each participant, fMRI was scheduled to avoid menstruation periods. Informed consent was obtained from all participants, and the protocol was approved by the Institutional Review Board (IRB) of Kyung Hee University Hospital at Gangdong (KHNMC-OH-IRB 2011-004). All experiments were conducted in accordance with the Declaration of Helsinki. One participant was excluded from the analysis because she was found to have multifocal T2 hyperintensive lesions in the subcortical white matter, which might be indicative of small vessel disease. The results of the remaining 19 participants were analyzed. None of the participants' head motion was greater than 5 mm. 

### 2.2. Definition and Manipulation of Acupoint BL60

The verum and sham acupuncture needling were applied at the same meridian BL60 acupoint (*KonLyun *in Korean, *KunLun* in Chinese) on the left [17]. It is located in the center of the depression between the prominence of the lateral malleolus of the fibula and the calcaneal tendon behind the ankle joint. For the verum acupuncture stimulation, a disposable stainless steel needle with a diameter of 0.25 mm and a length of 30 mm (DB106 Spring Handle, Dongbang Acupuncture Inc., Boryeong, Chungnam, Korea) was used. The verum acupuncture needle was inserted into the skin to a vertical depth of 1.0–1.5 cm in the deep tissue layer. For the sham acupuncture stimulation, a disposable placebo needle made of tungsten with a diameter of 0.25 mm and a length of 40 mm (Dongbang Acupuncture Inc., Boryeong, Chungnam, Korea) was used. The manipulation method consisted of constant tactile stimulation with a nonpenetrating needle to mimic acupuncture procedures. This procedure was performed by an experienced Korean medicine doctor and was conducted by the same person at each session.

### 2.3. Experimental Paradigms

**Figure 1:**
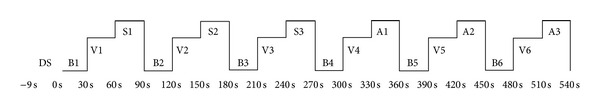
Block design of stimulations. A dummy scan was performed for 9 seconds before the experiment. Participants received alternating visual stimulations of the 30-second baseline video of the left leg (B) and the 30-second video of acupuncture treatment at the left BL60 (V). They then proceeded to undergo the 30-second sham acupuncture stimulation at BL60 with the same visual stimulation of the left leg (S) for 270 seconds followed by 30 seconds of B, 30 seconds of V, and 30 seconds of verum acupuncture at BL60 with the same visual stimulation of the left leg (A) for 270 seconds. DS: dummy scan, B: baseline stimulation, V: visual stimulation, S: sham acupuncture, A: verum acupuncture.

Each study participant underwent four sessions in this study. The order of the four sessions included sham and verum acupuncture stimulations prior to receiving local anesthesia, followed by the same stimulations after receiving local anesthesia (sham-verum-anesthesia-sham-verum).  Figure 1 shows the block design of fMRI paradigm for stimulation. At baseline, a 30-second video clip of a leg was shown as visual stimulation (B). Visual stimulation then included showing a video clip of verum acupuncture treatment performed on the leg, which had been prepared beforehand (V). Each participant watched the video clip using a mirror inside the MRI machine and was asked to concentrate on the visual stimulation and not to sleep, move, or think of anything else. Sham or verum acupuncture stimulation of 30 seconds was performed at the BL60 acupoint in addition to showing the same video clip of a leg (S or A). The needle was manually rotated clockwise and counterclockwise at a frequency rate of twice per second (2 Hz). Thus, the block-designed paradigm for the sham subsession was B-V-S-B-V-S-B-V-S, and for the verum sub-session was B-V-A-B-V-A-B-V-A.

After the first set of sham and verum sessions was completed, 0.2 mL of lidocaine HCl solution (Lidocaine HCl 2%/20 mL, Huons, Jecheon, Republic Korea) was injected into the region of acupoint BL60 as a local anesthetic agent. After obtaining an anatomic image to confirm the spot, the effects of local anesthesia were tested by pricking and performing the second set of sham and verum sessions using the same techniques previously described. After the fMRI session was completed, participants were asked to recall and rate their pain intensity.

### 2.4. fMRI Acquisition

All fMRI images were acquired using a 3.0 Tesla whole body MR scanner (Acheiva, Philips Medical System, Best, The Netherlands) with an 8-channel head coil. Participants were placed into the magnet bore head first in a supine position, wearing helmets lined with soft foam padding to help reduce motion artifact. 

To maximize the effect of BOLD, a gradient-echo planar imaging (EPI) sequence was used with the following parameters: imaging matrix size, 64 × 64 reconstructed to 96 × 96 pixels; field of view (FOV), 230 × 230 mm; echo time (TE), 35 ms; repetition time (TR), 3000 ms; and flip angle, 90°; 25 slices 5 mm thick, without a gap between slices; voxel size, 3.6 mm × 3.6 mm × 5.0 mm reconstructed to 2.4 mm × 2.4 mm × 5.0 mm, and total of 180 dynamic scans. 

Furthermore, anatomical images were acquired with a 2-dimensional (2D) T2-weighted turbo spin echo sequence (TR, 3000 ms; TE, 80 ms; flip angle, 90°; FOV, 230 × 230 mm; slice thickness, 3 mm; matrix size, 256 × 256; voxel resolution, 0.9 mm × 0.9 mm × 3.00 mm, and transverse orientation) and a 3-dimensional (3D) T1-weighted (T1W) gradient echo sequence (TR, 9.9 ms; TE, 4.6 ms; flip angle, 8°; FOV, 240 × 240 mm; slice thickness, 1 mm; matrix size, 240 × 240; voxel resolution, 1.0 mm × 1.0 mm × 1.0 mm, and sagittal orientation). The anatomical scan was performed shortly after local anesthesia using a lidocaine HCl injection into BL60. The total time for 2D and 3D anatomical imaging was 9 minutes and 47 seconds. 

### 2.5. Processing of fMRI Data

fMRI data analysis was performed using Statistical Parametric Mapping software (SPM5; Wellcome Department of Cognitive Neurology, London, UK; http://www.fil.ion.ucl.ac.uk/spm/) by way of MATLAB (Math Works Inc., Natick, MA). Head motion during the fMRI acquisition was calculated by rotation and translation on *x*,   *y*, and *z* coordinates and realigned automatically using three-dimensional motion correction to minimize the movement-related variance of the subject. Subjects whose heads moved excessively (>5 mm) were excluded from the analysis. Realigned anatomical MR images and fMRI images for each participant were coregistered to the 3D-T1W image. The 3D-T1W image and fMRI data were spatially normalized into the SPM Montreal Neurological Institute (MNI) template. Anatomical 3D MR images were standardized using standard anatomical space devised by Talairach and Tournoux, and fMRI images were standardized with the same process as the anatomical images. The spatially normalized image volumes were smoothed using a Gaussian kernel of the full width at half maximum (FWHM) of 8 × 8 × 10 mm to raise the power of discrimination among noises of MR signal changes classified by voxels produced by hemodynamic response and to set match statistical model offered by SPM. 

### 2.6. Statistical Analysis of fMRI Data

For the individual level analysis, in order to obtain activated or deactivated contrast maps, the stimulation conditions were compared to the baseline conditions for each session and each subject using a general linear model. For the sham subsession, the following contrast maps were obtained:

- sham acupuncture (S) > baseline (B), which were greater during the sham acupuncture stimulations than during the baseline visual stimulation before local anesthesia,
- sham acupuncture (S) < baseline (B), contrariwise.

For the verum acupuncture subsession, the following contrast maps were obtained:

(iii) acupuncture (A) > baseline (B) for activation maps,
(iv) acupuncture (A) < baseline (B) for deactivation maps.

The contrast maps after local anesthesia were also obtained in the same way. 

For the group level analysis, the activation or deactivation contrast maps obtained from the individual level analysis were used. In order to investigate the BOLD signal effect for each session, a one-sample *t*-test was performed for the 8 different contrast maps with a statistical threshold of *P* = 0.001, without multiple compensations, and with a cluster size ≥10 voxels. The result areas were limited to the grey matter with an explicit mask of grey matter (expression threshold: IL > 0.5). 

To investigate the pain-related effects and pain-unrelated effects, the differences between the pre- and post-anesthetic BOLD signal effects were also compared using a paired *t*-test. To investigate the acupuncture-specific or sham-specific effects, the sham and verum acupuncture stimulation blocks in each pre- and post-anesthetic session were included. 

### 2.7. Questionnaire of Self-Reported Sensations

Self-reported sensation was documented immediately after the MRI acquisition using a 0 to 10 numeric rating scale (NRS). To verify the successfulness of blinding, a self-reported guess of what happened during the visual stimulation of acupuncture (V) when no acupuncture was actually performed was also used.

## 3. Results

### 3.1. Areas of Activation and Deactivation of Each Session

**Figure 2:**
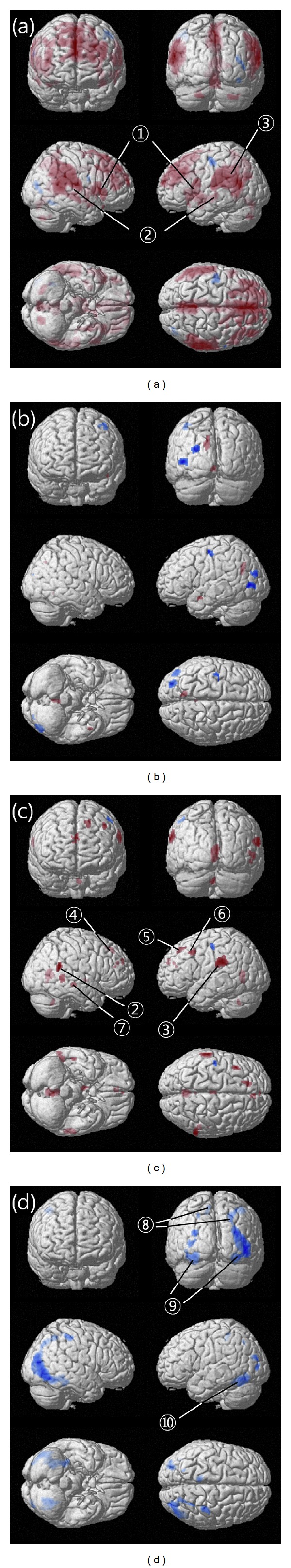
Brain activity during the sham or verum sessions before and after anesthesia. (a) Activated and deactivated brain areas during the sham session before anesthesia. (b) Activated and deactivated brain areas during the sham session after anesthesia. (c) Activated and deactivated brain areas during the verum session before anesthesia. (d) Activated and deactivated brain areas during the verum session after anesthesia. *①* Inferior frontal gyrus (R, L), *②* superior temporal gyrus (R, L), *③* inferior parietal lobule (L), *④* medial frontal gyrus (R), *⑤* superior frontal gyrus (L), *⑥* middle frontal gyrus (L), *⑦* middle temporal gyrus (R), *⑧* postcentral gyrus (L, R), *⑨* middle occipital gyrus (L, R), *⑩* inferior temporal gyrus (L). R: right, L: left. Activated areas are expressed in red, and deactivated areas are expressed in blue. Baseline stimulation is showing a video clip of a leg.

The differences in BOLD signals between the sham acupuncture block (S) and the baseline stimulation block (B) and between the verum acupuncture block (A) and the baseline stimulation block (B) were investigated; this was considered the additional change from the baseline.  Table 1 shows different activated/deactivated areas between the pre- and post-anesthetic sham acupuncture.  Table 2 shows different activated/deactivated areas between the pre- and post-anesthetic verum acupuncture (Figure 2).

### 3.2. Results of Self-Reported Sensations

Table 3 shows the type of sensation and mean (standard deviation) of each NRS score reported by study participants.

## 4. Discussion

In this study, differences between pain-related and pain-unrelated brain activation during acupuncture stimulation at the left BL60 were investigated. Pre-anesthetic brain activity included pain response, and post-anesthetic brain activity was related to pain-unrelated responses. To verify neuronal-specific effects of acupuncture, the differences between acupuncture sessions and baseline sessions (visual stimulation) were assessed. Therefore, the activated/deactivated brain areas discussed were presumed to be “acupuncture-specific effects only,” which are distinct from accessory effects such as anticipation, imagination, emotion, concentration, interaction with the doctor, and stimulation from the supine position. These accessory factors have been argued in previous studies [15, 18].

Verum or sham acupuncture at BL60 activated some regions in the frontal, temporal, parietal, and limbic lobes of the cortex and cerebellum before local anesthesia. It is thought that sensory inputs by acupuncture stimulation are transferred to the frontal cortex through long-range cortical tracts, and the regions in the frontal cortex send and receive signals with the cingulate gyrus and thalamus. 

In activated areas associated with pre-anesthetic verum or sham acupuncture stimulations, the inferior parietal lobule (IPL) and superior temporal gyrus (STG) have been involved in the perception of emotions in facial stimuli and the interpretation of sensory information [19, 20]. More specifically, the superior frontal gyrus is involved in self-awareness and coordinating actions with the sensory system [21]. The middle frontal gyrus, especially Brodmann area (BA) 8 including the frontal eye fields, is believed to play an important role in the control of eye movements. BL60 acupoint has been shown to be involved in visual information processing [22].

Moreover, pre-anesthetic verum acupuncture involved activation of the medial frontal gyrus associated with high-level executive functions and decision-related processes [23], as well as the tonsil or culmen, where ipsilateral eye movement is controlled unconsciously.

Brain areas that were more activated in pre-anesthetic verum than in sham procedures included the cingulate gyrus and thalamus, suggesting an association with the known pain transmission pathway [24] or the unpleasantness transmission pathway [25].

On the other hand, the postcentral gyrus was deactivated by verum or sham acupuncture before anesthesia. It is located in the primary somatosensory cortex (S1), the main sensory receptive area for the sense of touch, and receives thalamocortical projections from sensory input fields. The deactivation area in the talairach coordinate (*x* = −38, *y* = −22, *z* = 48  and  *x* = −52, *y* = −18, *z* = 50) is where the S1 somatotopic eye representation may exist [26] indicating that acupuncture at BL60 may control eye sensations, in addition to the sense of vision. The results above show the effects of acupuncture at BL60 including pain response. However, involvement of other areas might imply that verum acupuncture is associated with higher coordination function including vision. 

Post-anesthetic verum acupuncture, which is considered to be a pain-unrelated mechanism, showed deactivation of the middle occipital gyrus, inferior temporal gyrus, precuneus, and superior parietal lobule. The occipital lobe is the visual processing center containing most of the anatomical region of the visual cortex. The inferior temporal gyrus, which is connected behind the inferior occipital gyrus, is one of the higher levels of the ventral stream of visual processing and may also be involved in face perception. The precuneus is also involved in visuospatial processing as well as episodic memory, and the superior parietal lobule is involved with spatial orientation and receives a great deal of visual input as well as sensory input from one's hand.

Therefore, these results confirm the specificity of acupoint BL60 as suggested in previous studies and suggest the clinical usefulness of BL60 in body reorientation via a pain-related or pain-unrelated pathway.

Self-reported sensation was stronger in verum acupuncture than in sham procedures in all categories, whereas brain activation appeared to be broader in sham acupuncture than in verum acupuncture. Even though needle insertion is more painful and induces more intense sensations, continuous tactile stimulation appears to activate a broader brain area. According to a previous fMRI study with acupuncture and superficial pricking, multiple distributed activation was noted in superficial pricking [13] (similar to the sham acupuncture in the present study). Our results suggest that broader brain activation of sham acupuncture may be associated with repetitive superficial pricking compared to verum acupuncture.

Previous studies reported that acupuncture manipulation was associated with decreased fMRI signals [11, 12]. Deactivation in such instances may be interpreted as the suppression of neuronal activity [27] due to the hemodynamic or metabolic effects of downregulation of accompanying neuronal inhibition [8]. The deactivation of limbic-cerebellar areas can be one of the effects of acupuncture [24]. The results of the present study are in agreement with previous fMRI studies of “acupuncture deactivation.”

Traditionally, the acupoint BL60 has been used for disorders of upper or lower extremity, low back, arthritis of the hock, soft tissue injuries, and for eye-related disorders such as visual dizziness, redness, swelling of the eyes, as well as bursting eye pain [6, 18]. Recent research showed that this acupoint activates bilateral regions within the visual cortex through interhemispheric visual-visual transmission, as well as adjacent regions in nonvisual cortices [16]. Additionally, tempospatial analysis showed vision-related acupoint specificity, including BL60 [15].

According to the present study, needling BL60 appeared to activate and deactivate various brain areas and was especially related to more vision control, as well as being involved in higher-order perception and fine regulation of emotion and information. The pain-unrelated response included deactivation of the middle occipital gyrus, postcentral gyrus, precuneus, superior parietal lobule, and cerebellar declive. The pain-related response included activation of the thalamus and cingulate gyrus, as well as superior and medial frontal gyri and middle temporal gyrus. Additionally, the left cerebellar culmen was commonly activated in the pre-anesthetic sham and verum acupunctures and post-anesthetic sham acupuncture, which might suggest a specific response to acupuncture with or without pain. 

## 5. Conclusions

Pre-anesthetic verum acupuncture at BL60 activated areas of the brain associated with vision, as well as pain transmission; these findings are tantamount to a pain-related acupuncture response. Post-anesthetic verum acupuncture at BL60 deactivated brain areas of visual function, which is considered to be a pain-unrelated acupuncture response. These results indicate that specific effects of acupoint BL60 are to control visual sense as used in the clinical setting.

## Figures and Tables

**Table 1:** Activated and deactivated brain areas during the sham acupuncture before and after anesthesia.

	Area (*x*, *y*, *z*)	Side	KE	*T* peak	*Z* score
Sham > base (pre-anesthetic)	Cortex				
	Frontal lobe, inferior frontal gyrus (−50,8, 16)	L	213	6.81	4.73
	Frontal lobe, inferior frontal gyrus (40,20,8)	R	685	7.49	4.98
	Temporal lobe, superior temporal gyrus BA 22 (−60, −58,16)	L	2341	10.87	5.97
	Temporal lobe, superior temporal gyrus BA 22 (66, −44,8)	R	3103	8.64	5.57
	Parietal lobe, inferior parietal lobule (−60, −32,34)	L	2341	7.05	4.82
	Limbic lobe, cingulate gyrus BA 24 (−4,12,28)	L	10039	9.13	5.51
	Sublobar, claustrum (−34,0, −4)	L	793	8.34	5.27
	Sublobar, caudate (−14,10,6)	L	533	6.91	4.77
	Cerebellum				
	Anterior lobe, culmen (−2, −60,4)	L	10039	9.07	5.49

Sham > base (post-anesthetic)	Cortex				
	Occipital lobe, precuneus (−14, −64,28)	L	54	4.63	3.71
	Temporal lobe, middle temporal gyrus (−46,2, −18)	L	10	4.61	3.70
	Cerebellum				
	Anterior lobe, culmen (0, −56, −8)	L	37	4.05	3.37

Sham < base (pre-anesthetic)	Cortex				
	Frontal lobe, precentral gyrus (−44, −14,56)	L	37	4.19	3.46
	Parietal lobe, postcentral gyrus BA 3 (−38, −22,48)	L	37	4.19	3.46

Sham < base (post-anesthetic)	Cortex				
	Occipital lobe, middle occipital gyrus	L			
	(−24, −88,20)		59	4.77	3.79
	(−48, −74,2)		63	4.46	3.62
	Frontal lobe, precentral gyrus BA 6 (−40, −12,58)	L	27	4.25	3.49

*P*_uncorrected_ (cluster level) < 0.001, cluster size ≥ 10 voxels.

Sham: sham acupuncture; base: baseline visual stimulation by showing a video clip of a leg; R: right; L: left; KE: expected voxels per cluster.

**Table 2:** Activated and deactivated brain areas during the verum acupuncture before and after anesthesia.

	Area (*x*, *y*, *z*)	Side	KE	*T* peak	*Z* score
Acup > base (pre-anesthetic)	Cortex				
	Frontal lobe, superior frontal gyrus	L			
	BA 8 (−16,34,48)		24	5.32	4.07
	(0,54,26)		12	3.82	3.23
	Frontal lobe, middle frontal gyrus BA 8 (−40,16,44)	L	33	5.80	4.30
	Frontal lobe, medial frontal gyrus (2,42,22)	R	14	3.77	3.20
	Parietal lobe, inferior parietal lobule BA 40 (−62, −34,30)	L	127	5.65	4.23
	Limbic lobe, posterior cingulate BA 40 (0, −62,8)	L	177	5.11	3.97
	Temporal lobe, superior temporal gyrus BA 22 (64, −48,20)	R	54	4.61	3.70
	Sub-lobar, thalamus (8, −10, −2)	R	17	4.41	3.59
	Temporal lobe, middle temporal gyrus	R			
	(54, −24, −8)		14	4.33	3.54
	(56, −42, −2)		31	3.97	3.32
	Limbic lobe, cingulate gyrus BA 24 (0, −22,36)	L	11	3.88	3.27
	Cerebellum				
	Anterior lobe, culmen (0, −52, −4)	L	35	4.64	3.72
	Posterior lobe, cerebellar tonsil (−2, −58, −38)	L	24	4.11	3.41

Acup > base (post-anesthetic)	None				

Acup < base (pre-anesthetic)	Cortex				
	Parietal lobe, postcentral gyrus BA 1 (−52, −18,50)	L	14	4.02	3.35

Acup < base (post-anesthetic)	Cortex				
	Occipital lobe, middle occipital gyrus (46, −72, −8)	R	1144	6.11	4.44
	Occipital lobe, middle occipital gyrus (−24, −88,20)	L	59	4.77	3.39
	Occipital lobe, inferior temporal gyrus (−48, −72,2)	L	63	4.46	3.62
	Parietal lobe, postcentral gyrus BA 40 (40, −32,54)	R	83	4.79	3.80
	Parietal lobe, precuneus BA 7 (−12, −40,52)	L	47	4.27	3.50
	Parietal lobe, superior parietal lobule BA 7 (−22, −66,46)	L	10	3.95	3.31
	Cerebellum				
	Posterior lobe, declive (−30, −64, −14)	L	427	6.60	4.65

*P*_uncorrected_ (cluster level) < 0.001, cluster size ≥ 10 voxels.

Acup: verum acupuncture; base: baseline visual stimulation by showing a video clip of a leg; R: right; L: left; KE: expected voxels per cluster.

**Table 3:** Self-reported sensations using a 0 to 10 numeric rating scale (NRS) and the mean (standard deviation) of each NRS score.

Type of sensation	Mean (standard deviation)		Differences of average rating (Verum-Sham)*	*P*-value
	Sham acupuncture	Verum acupuncture		
The moment when the needle touches the skin				
Hurting	1.15 (2.39)	2.95 (2.74)	1.80	0.002*
Penetrating	3.40 (2.64)	5.65 (2.50)	2.25	0.002*
Sharp pain	2.65 (1.83)	4.90 (2.85)	2.25	<0.001*
Aching	1.75 (2.02)	3.95 (2.74)	2.20	<0.001*
Subtotal average	2.24	4.36	2.12	<0.001*

Throughout the acupuncture stimulation				
Aching	2.10 (2.18)	3.80 (2.07)	1.70	<0.001*
Soreness	1.65 (1.95)	2.80 (2.78)	1.15	0.013*
Deep pressure	1.70 (2.20)	3.95 (2.61)	2.25	<0.001*
Heaviness	2.25 (1.70)	3.85 (2.68)	1.60	0.004*
Fullness (distention)	1.45 (2.48)	3.50 (2.46)	2.05	<0.001*
Tingling	2.35 (1.34)	4.25 (3.35)	1.90	0.014*
Warmth	0.70 (1.80)	1.15 (2.21)	0.45	0.119
Cold	1.25 (2.26)	1.65 (2.32)	0.40	0.258
Numbness	2.20 (1.84)	2.40 (3.02)	0.20	0.725
Dull pain	2.00 (1.64)	2.80 (2.82)	0.80	0.084
Throbbing	1.05 (2.14)	2.10 (2.63)	1.05	0.011*
Sharp pain	2.20 (2.13)	4.35 (3.41)	2.15	0.005*
Spread	2.10 (2.88)	3.25 (2.83)	1.15	0.034*
Anxiety	2.75 (1.38)	4.75 (3.39)	2.00	0.002*
Other sensation	0.70 (1.52)	1.40 (2.39)	0.70	0.090
Subtotal average	1.76	3.07	1.31	<0.001*

Total average	1.86	3.34	1.48	<0.001*

For each sensation, a paired *t*-test was performed to compare the score between the sham and the verum acupuncture stimulation.

*P < 0.05.
